# Differential Cytokine Changes in Patients with Myasthenia Gravis with Antibodies against AChR and MuSK

**DOI:** 10.1371/journal.pone.0123546

**Published:** 2015-04-20

**Authors:** Vuslat Yilmaz, Piraye Oflazer, Fikret Aysal, Hacer Durmus, Kostas Poulas, Sibel P. Yentur, Yesim Gulsen-Parman, Socrates Tzartos, Alexander Marx, Erdem Tuzun, Feza Deymeer, Güher Saruhan-Direskeneli

**Affiliations:** 1 Department of Physiology, Istanbul Medical Faculty, Istanbul University, Istanbul, Turkey; 2 Department of Neurology, Istanbul Medical Faculty, Istanbul University, Istanbul, Turkey; 3 Department of Neurology, Bakirkoy Research and Training Hospital for Psychiatric and Neurological Diseases, Istanbul, Turkey; 4 Department of Pharmacy, School of Health Sciences, University of Patras, Patras, Greece; 5 Hellenic Pasteur Institute, Athens, Greece; 6 Institute of Pathology, University Medical Centre Mannheim, University of Heidelberg, Mannheim, Germany; 7 Department of Neuroscience, Institute for Experimental Medical Research, University of Istanbul, Istanbul, Turkey; Georgia Regents University, UNITED STATES

## Abstract

Neuromuscular transmission failure in myasthenia gravis (MG) is most commonly elicited by autoantibodies (ab) to the acetylcholine receptor or the muscle-specific kinase, constituting AChR-MG and MuSK-MG. It is controversial whether these MG subtypes arise through different T helper (Th) 1, Th2 or Th17 polarized immune reactions and how these reactions are blunted by immunosuppression. To address these questions, plasma levels of cytokines related to various Th subtypes were determined in patients with AChR-MG, MuSK-MG and healthy controls (CON). Peripheral blood mononuclear cells (PBMC) were activated *in vitro* by anti-CD3, and cytokines were quantified in supernatants. In purified blood CD4^+^ T cells, RNA of various cytokines, Th subtype specific transcription factors and the co-stimulatory molecule, CD40L, were quantified by qRT-PCR. Plasma levels of Th1, Th2 and Th17 related cytokines were overall not significantly different between MG subtypes and CON. By contrast, *in vitro* stimulated PBMC from MuSK-MG but not AChR-MG patients showed significantly increased secretion of the Th1, Th17 and T follicular helper cell related cytokines, IFN-γ, IL-17A and IL-21. Stimulated expression of IL-4, IL-6, IL-10 and IL-13 was not significantly different. At the RNA level, expression of CD40L by CD4^+^ T cells was reduced in both AChR-MG and MuSK-MG patients while expression of Th subset related cytokines and transcription factors were normal. Immunosuppression treatment had two effects: First, it reduced levels of IL12p40 in the plasma of AChR-MG and MuSK-MG patients, leaving other cytokine levels unchanged; second, it reduced spontaneous secretion of IFN-γ and increased secretion of IL-6 and IL-10 by cultured PBMC from AChR-MG, but not MuSK-MG patients. We conclude that Th1 and Th17 immune reactions play a role in MuSK-MG. Immunosuppression attenuates the Th1 response in AChR-MG and MuSK-MG, but otherwise modulates immune responses in AChR-MG and MuSK-MG patients differentially.

## Introduction

Myasthenia gravis (MG) is an acquired autoimmune disease caused by a dysfunction at the postsynaptic part of the neuromuscular junction (NMJ). The disease is heterogeneous with respect to age at onset, thymic changes and presence of auto-antibodies (ab) [1]. The symptoms of MG are mediated by pathogenic ab directed mainly against the nicotinic acetylcholine receptor (AChR) in 80–90% of patients (AChR-MG), leading to reduced numbers of AChR molecules at the postsynaptic end-plates of the NMJ [2–4]. The AChR-specific ab include predominantly complement-fixing isotypes such as the immunoglobulin (Ig) IgG1 and IgG3 [5] and exert their effects mainly by activation of the complement cascade [6,7]. Approximately 40–70% of the small subgroup of anti-AChR ab negative MG patients has predominantly non-complement fixing IgG4 ab against muscle specific kinase (MuSK) (MuSK-MG) [8–10]. The patients without either anti-AChR or anti-MuSK ab make up the double-seronegative group (SN-MG), over 60% of whom have shown to display low affinity anti-AChR ab [11]. Recently, ab directed against low density lipoprotein receptor-related protein 4 (LRP4) and agrin have been reported mainly in double seronegative cases [12–16].

Cognate interaction between T and B lymphocytes is essential for the production of high-affinity antibodies and for long-term immunologic memory. B cells maturing to produce ab in autoimmune diseases largely require T cell help, which is mainly coordinated by cytokines from T helper (Th) cells. Several studies have demonstrated that mostly Th1 type and/or proinflammatory T cells are involved in anti-AChR ab production, also in the experimental animal model of MG [17–22]. Deficiency of cytokines in experimental animals often leads to significant reduction in AChR antibody production and experimental autoimmune myasthenia gravis (EAMG) susceptibility [23]. However, very little is known about the immunologic factors that underlie the generation of anti-MuSK antibodies. While a single recent study has demonstrated that MuSK-MG patients have higher frequencies of Th1 and Th17 type cells than normal controls [24], an experimental model study untreated animals showed that MuSK immunization preferentially promoted the production of Th2 type cytokines as IL-4 and IL-10 [25].

To delineate the characteristic cytokine profiles of MuSK-MG patients and find out cytokine production patterns that might potentially differentiate MuSK-MG and AChR-MG patients, we compared cytokine responses in two different MG patient groups at the same time with healthy controls (CON). Cytokines were evaluated ex vivo by protein measurements in plasma and in culture supernatants of non-stimulated peripheral blood mononuclear cell (PBMC), as well as by mRNA measurements in isolated CD4^+^ T cells. To provoke differences, non-specific T cell stimulation was applied on PBMC to see differential inducible responses of T cell derived cytokines.

## Materials and Methods

### Study group

The patients were clinically diagnosed as having MG and classified by the presence of anti-AChR and anti-MuSK ab, which were screened using radioimmunoassay (RIA) with commercial kits (DLD Diagnostika GmBH, Hamburg, Germany). All patients had generalized non-thymomatous disease. Among the patients included in the study, 69% of those who donated blood for peripheral blood mononuclear cell (PBMC) culture, 58% of the patients’ whose RNA was used for expression analysis, and 51% of patients’ whose plasma cytokine levels were measured, were receiving immunosuppressive (IS) treatment (prednisolone and/or azathioprine). Among the 23 MuSK-MG patients 4 did not receive immunosuppression. AChR-MG and MuSK-MG patients were managed with the same treatment algorithm. Controls (CON) were recruited from the healthy population with no known disease and matched with the MG group for age and sex distribution. The distribution of patient groups and healthy controls included in different tests are summarized in Table 1.

**Table 1:** Demographic characteristics of the donors in anti-AChR ab positive (AChR-MG), anti-MuSK ab positive (MuSK-MG) MG patient and healthy control (CON) groups.

		AChR-MG	MuSK-MG	CON
Plasma	Total (N)	46	23	42
	Women/Men	26/20	16/7	15/27
	Mean Age	42.2± 16.7	40.5 ± 17.7	40.2± 10.6
	Treatment (yes/no)	16/30	19/4	
PBMC culture	Total (N)	29	13	15
	Women/Men	17/12	9/4	9/6
	Mean Age	46.2± 17.1	44.4± 8.7	44.5± 16.6
	Treatment (yes/no)	18/11	11/2	
mRNA expression	Total (N)	27	18	26
	Women/Men	16/11	12/6	13/13
	Mean Age	38± 18.4	43.3± 18	39.2± 11.1
	Treatment (yes/no)	11/16	15/3	

PBMC, peripheral blood mononuclear cells.

Treatment included only immunosuppressive (IS) agents.

The Ethical Review Board of Istanbul Medical Faculty approved the study and written informed consent was obtained from the patients before blood donation.

### Isolation of PBMC and cell culture

PBMC were separated by using standard Ficoll density gradient centrifugation (Sigma). After washing twice, viable cells were counted with trypan blue and incubated in culture medium at 2x10^5^ cells/well in plates at 37°C containing 5% CO_2_. Culture medium in RPMI-1640 (Sigma) was supplemented with 2 mM L-glutamine (Sigma), 100 IU/100 mg/ml penicillin/streptomycin (Sigma) and 10% fetal bovine serum (Gibco). Although in preliminary experiments, PBMC were stimulated specifically with AChR and MuSK, no appreciablecytokine production could be achieved (data not shown). Considering T cells as the major effectorsin MG pathogenesis, we used the non-specific T cell stimulator against the CD3. Plate-bound anti-CD3 antibody (Biolegend) was used with a final concentration of 2 μg/ml. After 72 hours, the supernatants of the cultures from duplicate wells were collected and stored at -80°C until analysis.

### Measurement of cytokines

IL-10, IL-17A, IFN-γ and IL-13 were measured in the culture supernatants using multiplex immunoassays (Millipore, Milliplex Map Kit). IL-21 (eBioscience), IL-4 (eBioscience, High Sensitivity) and IL-6 (Peprotech) were detected using ELISA in accordance with the manufacturer’s protocol. IFN-γ, IL-10, IL-12p40, IL-13 and IP-10 (CXCL10) levels were determined in patients’ plasma samples (Millipore, Milliplex Map Kit). The levels of the cytokines were quantified by reference to standard curves. As the standards were not covering all values obtained from the samples, median values of the readings in “Luminex” and optic density measurements in ELISA were used for the statistical analysis. Results were expressed as pg/ml and ng/ml in the figures. In the PBMC cultures, the induced levels for each cytokine were calculated by subtracting the cytokine levels in the supernatants of non-stimulated wells from those that were CD3-stimulated.

#### RNA quantification of expressed cytokines and transcription factors

CD4^+^ T cells were purified from PBMC using magnetic cell isolation beads according to the manufacturer’s instructions (MACS, Miltenyi Biotech). CD4^+^ T cell purity was >98% by flow cytometry. RNA was extracted from the isolated CD4^+^ T cells using an RNA isolation kit (RNeasy Mini Kit, Qiagen). mRNA levels of transcription factors (*TBET*, *GATA3*, *RORC*, *PRDM1)*, cytokines (*IFNG*, *IL10*, *IL17A*, *IL21)* and *CD40L* were determined semi-quantitatively using real-time polymerase chain reaction (RT-PCR) with Sybr Green. RNA was reverse transcribed using Moloney Murine Leukemia virus reverse transcriptase (M-MuLV, Fermentas) with random hexamer primers (Roche) and the obtained cDNA was used for the quantification of mRNA levels. The PCR reactions were optimized with specific primer sets and performed on the LightCycler (FastStart DNA Master SYBR Green I, Roche Light Cycler 480 II) (S1 Table). Relative quantification of target genes was performed by 2^–ΔΔCT^ method using the glyceraldehyde 3-phosphate dehydrogenase (GAPDH) as the reference gene.

#### Statistical analysis

Differences between the patient and control groups and differences between the effects of stimulation by anti-CD3 antibody were tested for significant differences using nonparametric Mann-Whitney U test. Results are presented as mean values and p values below 0.05 were considered significant (two tailed). Statistical analyses and graphs were performed using SPSS (21.0) and GraphPad Prism (5.01) software.

## Results

### Cytokine production of PBMC in AChR-MG and MuSK-MG

**Fig 1:**
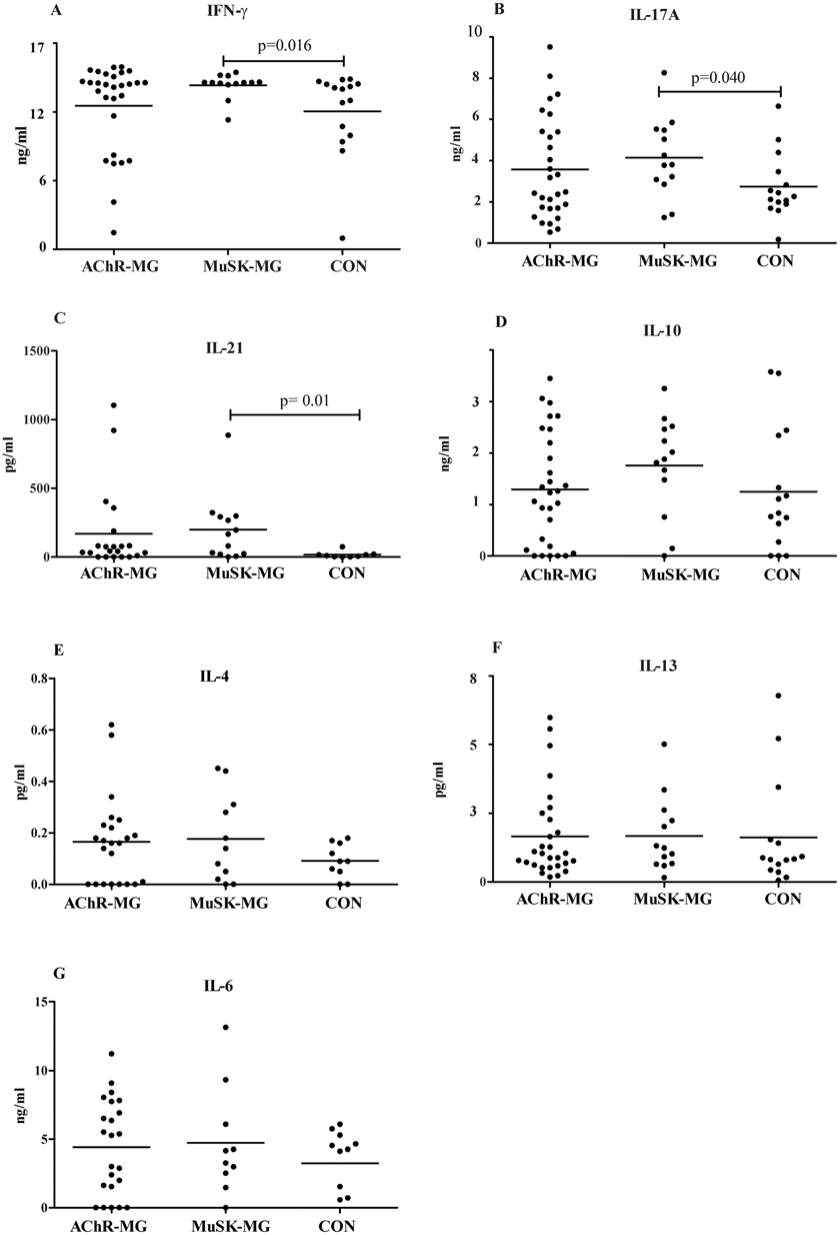
Cytokine production of PBMC in response to CD3 stimulation. A) IFN-γ, B) IL-17A, C) IL-21, D) IL-10, E) IL-4, F) IL-13 and G) IL-6 levels in the PBMC cultures from AChR-MG, MuSK-MG and CON groups. Horizontal lines indicate mean values.

To investigate the Th subset-mediated immune responses modulating anti-AChR and anti-MuSK antibody generation, cytokine production levels of anti-CD3 stimulated PBMC were compared among patient and CON groups. Following CD3 stimulation, MuSK-MG patients showed higher Th1 related IFN-γ, Th17 derived IL-17A and T follicular helper (Tfh) associated IL-21 production than CON (p = 0.016, p = 0.04 and p = 0.01, Fig 1A–1C). Although the levels of IFN-γ, IL-17A and IL-21 were also relatively higher in AChR-MG patients, the differences to the CON did not reach statistical significance. Moreover, MuSK-MG patients showed trends towards exhibiting higher levels of IL-10 and IL-4 in the supernatants. However, these differences did not attain statistical significance (Fig 1D and 1E). IL-13 and IL-6 cytokine responses were also not significantly different between disease subgroups (Fig 1F and 1G).

### Plasma cytokine levels of AChR-MG and MuSK-MG patients

To delineate Th cell related differences between AChR-MG, MuSK-MG and CON groups ex vivo, IFN-γ, IL-13, IL-10 and IL-17A levels were also determined in plasma samples. Beneath IL-10, preferential induction of IgG4 isotype has been reported for IL-12 [26,27]. IL12p40 levels were also evaluated as the common chain of both proinflammatory IL-12 and IL-23 which may lead to Th1 and Th17 immunities. As an inflammatory chemokine promoting activated T cell chemotaxis, IP-10 (CXCL10) was also measured. The levels of these cytokines and the chemokine were not significantly different between the groups (S2 Table).

### Expression of cytokines in CD4^+^ T cells of AChR-MG and MuSK-MG patients

To further evaluate possible alterations in Th1, Th2, Th17 and Tfh cell subsets, the expression of Th subtype related genes were measured in isolated CD4^+^ T cells. The mRNA levels of respective cytokines of the T cell subsets, namely *IFNG*, *IL10*, *IL17A* and *IL21* and the related transcription factors (*TBET*, *GATA3*, *RORC* and *PRDM1)* in CD4^+^ T cells were similar in AChR-MG, MuSK-MG and CON groups (S3 Table). No evidence for a differential polarization for Th1, Th17, Th2 and Tfh cells was detected in the expression analysis in disease subgroups at the RNA level.

**Fig 2:**
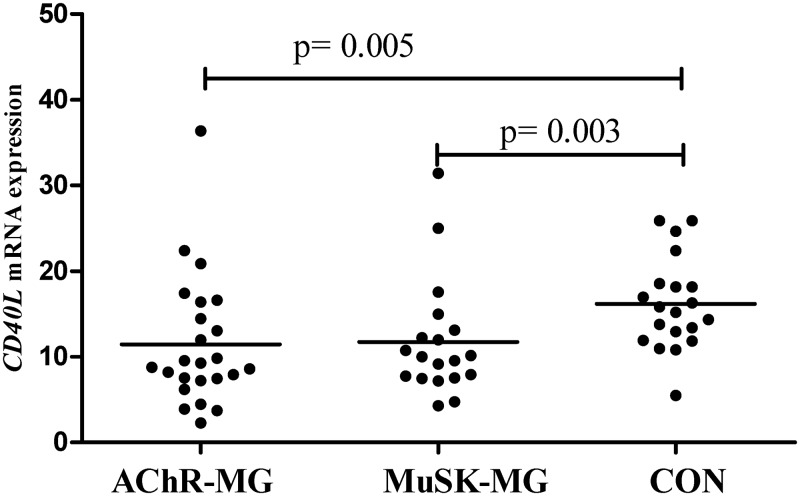
*CD40L* mRNA levels in the isolated CD4^+^ T cells. Expression levels from both treated and untreated patients in AChR-MG, MuSK-MG and CON groups are shown. Horizontal lines indicate mean values.

As an indispensable costimulatory molecule for specific ab production by B cells, CD40L mRNA expression was measured in freshly isolated CD4^+^ T cells as well. Expression of *CD40L* was lower both in MuSK-MG and AChR-MG groups compared with CON (p = 0.005 and p = 0.003, Fig 2).

### Effects of IS treatment on cytokines

**Fig 3:**
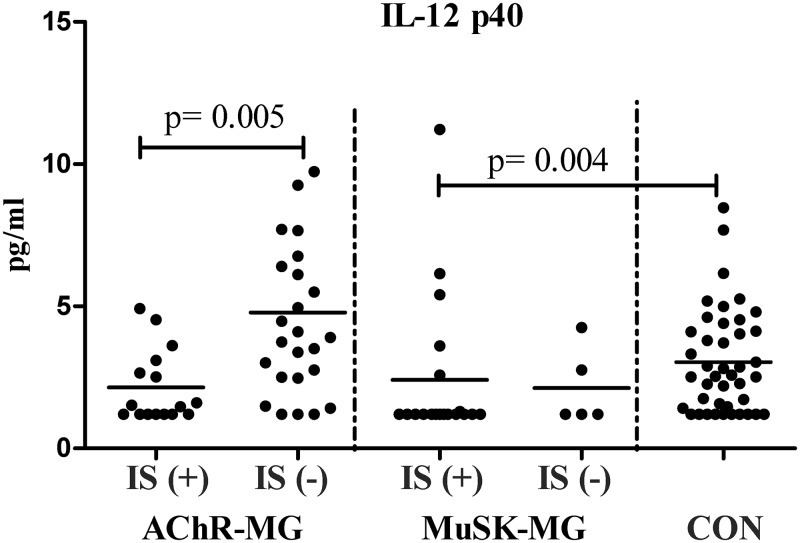
Plasma IL-12p40 levels. Groups of patients with AChR-MG, MuSK-MG under immunosuppressive treatment [IS (+)], untreated [IS (-)] and CON are shown. Horizontal lines indicate mean values.

The effects of IS were evaluated separately in plasma and cell culture supernatants, because a considerable proportion of the patient groups were on treatment. A decrease in plasma IL-12p40 levels was observed in IS treated AChR-MG patients (n: 16) compared with untreated (n: 25) patients (p = 0.005), whereas the difference to CON was not statistically significant (Fig 3). A similar effect was also detected in IS treated MuSK-MG patients (n: 19), who had significantly lower IL-12p40 levels than CON (p = 0.004, Fig 3). This effect was found out to be more pronounced when the entire MG cohort (both AChR- and MuSK-Ab positive patients) was analyzed. IS treated MG patients had lower plasma levels of IL-12p40 (1.5 pg/ml) than non-treated MG patients (3.6 pg/ml, p < 0.001) and CON (2.5 pg/ml, p = 0.006). Plasma levels of IL-13, IL-10, IFN-γ and IL-17A were not significantly different between these subgroups (not shown).

**Fig 4:**
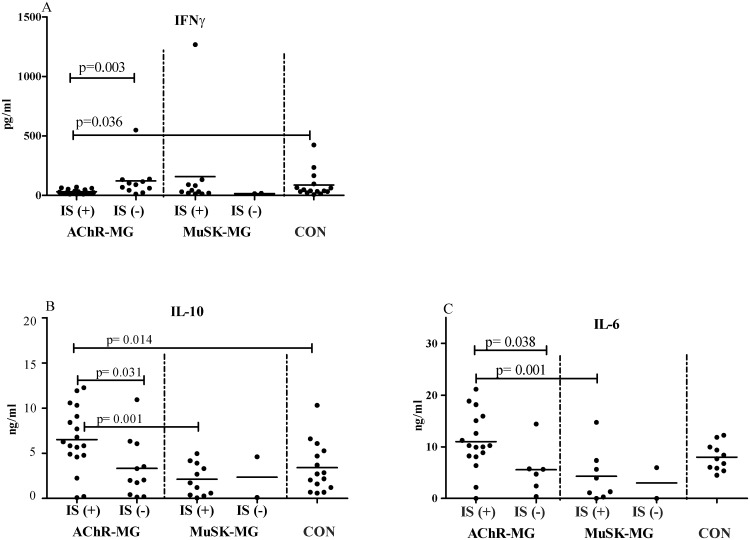
Spontaneous production of cytokines in culture. A) IFN-γ, B) IL-10 and C) IL-6 levels in PBMC cultures from AChR-MG, MuSK-MG patients under immunosuppressive treatment [IS (+)], untreated [IS (-)] and CON groups. Horizontal lines indicate mean values.

Treatment effect was also evaluated in terms of spontaneous cytokine production by PBMC. PBMC of IS-treated AChR-MG patients showed significantly reduced spontaneous IFN-γ production as compared to those of non-treated AChR-MG patients (p = 0.003) and CON (p = 0.036, Fig 4A), suggesting that Th1 type immune responses were depressed by IS treatment. A similar reduction in IFN-γ production was not observed in the MuSK-MG group. Conversely, spontaneous IL-10 production was increased in IS-treated AChR-MG patients (n: 18, p = 0.031) with IL-10 levels being even higher than the CON (p = 0.014, Fig 4B). IL-10 secretion of PBMC in IS-treated AChR-MG patients was also higher than that of IS-treated MuSK-MG patients (n: 11, p = 0.001) implicating that this difference is AChR-MG subgroup related. Similarly, in the AChR-MG group, IS-treated patients (n: 17) showed higher IL-6 production than non-treated (n: 6, p = 0.038) and treated MuSK-MG patients (n: 11, p = 0.001, Fig 4C). Comparisons between treated and non-treated patients could not be performed in MuSK-MG because only 2–4 patients without IS treatment could be included in the cytokine-production assays. However, IS treatment did not have an inducing effect on IL-10 and IL-6 secretions in treated MuSK-MG patients and the production of these cytokines stayed lower than the IS-treated AChR-MG group. Spontaneous production of IL-4, 17A, IL-13 and IL-21 by PBMC did not differ between treated and non-treated patients.

## Discussion

In this study, cytokines as modulators of differential ab production were evaluated in MuSK-MG and AChR-MG patients at several levels. Plasma cytokine levels and cytokine production patterns of cultured PBMC and CD4^+^ T cells were assessed and compared among MG subgroups and CON. Since under basal non-stimulated conditions, ex vivo protein and mRNA levels of Th related cytokines were not different between MG subgroups and CON, cytokine production levels of stimulated PBMC were compared. There were no differences among cytokine production patterns of PBMC specifically stimulated with MuSK or AChR (data not shown). Therefore, we used the non-specific T cell stimulant, anti-CD3, which revealed differential IL-17A, IFN-γ and IL-21 production patterns in different MG subgroups. Following T cell stimulation, IL-17A, IFN-γ and IL-21 productions were prominently increased in MuSK-MG patients, whereas this effect was not as strong in AChR-MG patients. This is the first study on comparison of cytokine production patterns of AChR-MG and MuSK-MG patients.

IL-17 and IL-21 are mainly produced by Th17 and Tfh cells and have been investigated in MG in only a few studies using heterogeneous MG groups. IL-17 has been reported to be increased in the sera (IL-17) and PBMC culture of patients with thymoma (IL-17A) only [28,29]. By contrast, in a more comprehensive recent evaluation, no IL-17 could be detected in sera of AChR-MG patients [30]. On the other hand, in MuSK-MG, immune response mechanisms associated with ab production have only been evaluated very recently. In this first study, proportions of CD4^+^ T cells with intracellular IL-17, IL-2 and TNF-α were found to be increased, while levels of IL-21 and IFN-γ positive cells were not altered [24]. In the present study, both IL-17A and IFN-γ levels were increased in MuSK-MG confirming the involvement of Th1 and Th17 type immune response in this disease subtype. More data are needed to clarify the role of IL-17A and IFN-γ to MuSK-MG.

As the cytokine of lymphoid germinal centers functioning at the T and B cell interaction, IL-21 was screened in a small study and reported to be increased in the sera of AChR-MG patients [31]. In our study, although IL-21 production levels of stimulated PBMC were increased in both MuSK-MG and AChR-MG groups, a higher magnitude of increase was observed in the MuSK-MG group. A previously reported induction of IgG4 secretion by IL-21 supports this observation in MuSK-MG [32]. Given the well-established requirement for T cell help for both anti-AChR and anti-MuSK ab production by B cells, further studies are warranted for determination of the mechanisms by which this Tfh derived cytokine is involved in MG pathogenesis.

So far, the only well-established and outstanding difference between the immunologic features of MuSK-MG and AChR-MG is the IgG isotype involved in MG pathogenesis. The non-complement fixing IgG4 is the predominant isotype in MuSK-MG, whereas complement activating IgG1 and IgG3 are predominantly found in sera of AChR-MG patients [5,11]. Both in mice and humans, non-complement fixing Ig isotypes are mainly regulated by Th2-type immunity (e.g. IL-4, IL-10), whereas complement-fixing isotypes are regulated by Th1 cytokines (e.g. IFN-γ) [27,33,34]. Previously, an increased Th2-type cytokine production was observed in MuSK immunized mice, consistent with the predominance of anti-MuSK IgG1 (equivalent of human IgG4) production [25]. In humans, however, Th2-type related pathways have not yet been studied in MuSK-MG. In the present study, in contrast with the animal experiments, a predominance of Th1- and Th17-related cytokines was observed in MuSK-MG patients. Nevertheless, in support of the previous animal model study, MuSK-MG patients’ stimulated PBMC produced relatively higher levels of IL-10 (a key cytokine in IgG4 production) than those of AChR-MG patients and CON (Fig 1D). This discrepancy between animal and human studies might be explained by different immunopathogenic factors in MG and experimental models of MuSK-MG. In the animal model of MG, immunologic factors were assessed immediately after the induction of disease and therefore observed differences might plausibly be more representative of the earlier stages of human MG. In human MG studies however, patients are assessed in relatively more advanced stages of MG, which might lead to introduction of additional confounding factors (medications, thymectomy etc.) that may alter Th responses. Moreover, in experimental studies, following MuSK immunization mice are sensitized to only a single antigen. By contrast, MuSK-MG patients may display a more complex immune response to MuSK leading to antigen diversification with isotypic variability including some IgG1 responses as well. It is also possible that the Th2 dominated T cell response is not prominently detectable at the peripheral blood of the MuSK-MG patients, whereas it may act at the tissue level.

Another main finding of this study was the demonstration of IS treatment’s down-regulating effect on Th1 cytokines, such as IFN-γ and IL12p40 and up-regulating effect on IL-10 and IL-6, which implicated a mechanism of treatment-induced functional regulation in AChR-MG. A similar effect of plasma exchange on IL-10 has been demonstrated previously and has been associated with good response to therapy [35]. The reported inhibitory effect of glucocorticoids on the shift of relative Th1/Th2 ratio towards Th1 could be confirmed mainly in AChR-MG in the present study [36]. However, MuSK-MG patients’ immune response to the same treatment was different. The up-regulating effect of IS treatment on IL-10 and IL-6 was not evident in MuSK-MG group differentiating this subgroup from AChR-MG. These differential cytokine production patterns under treatment may also contribute to the recent observation of relative resistance to IS treatment and better response to rituximab treatment in MuSK-MG patients [37].

Cognate interaction of CD40L on T cells with CD40 on B cells induces initiation of immunoglobulin isotype switch. Apart from the cytokines, the CD40L/CD40 co-stimulation is an important regulator of Th1 responses. CD40-CD40L interaction could have regulating effects on subgroup specific ab production, since ab production is T cell-dependent in MG. Down-regulation of Th1 mediated EAMG by blockade of CD40L has also implicated the effect of this interaction on Th1 type cytokines [38]. Co-stimulatory pathways have previously been investigated in MG and low percentages of CD40L^+^CD4^+^ and CD40L^+^CD8^+^ T cell populations have been found in peripheral blood samples of AChR-MG patients [39]. Likewise, in our study, CD40L expression of CD4^+^T cells of both MG subgroups was found even lower than CON with no difference between MuSK-MG and AChR-MG. Previous reports also demonstrated that maintenance of CD27^+^ B cells is favored when relatively lower levels of CD40L molecules were available for B cells [40]. This finding is in accordance with our previous finding of increased memory B cells (CD19^+^CD27^+^) in patients with MG [41] and may be explained by regulatory circuit of T-B cells resulting in a long-term maintenance of memory B cells.

Cytokine measurements in the plasma and at the mRNA levels in CD4^+^ T cells did not reveal any significant differences between disease groups and controls. A limitation of this approach could be the high proportion of IS-treated patients in both AChR-MG and MuSK-MG groups. However, no IS effect could be demonstrated in our study contradicting this argument. Previous cytokine expression studies performed in more heterogeneous MG patient populations with a high proportion of AChR-MG group were also unable to demonstrate any alterations in the sera [22,42]. As a chronic and not highly inflammatory environment, isolated CD4^+^ T cells from peripheral blood was also not informative enough for the cytokine activities in MG. Cytokines at the mRNA level have previously been studied in PMBC and the levels of TNF-α and IL-10 were found to be decreased in MG. Neither the presence of anti-AChR ab nor IS-treatment was significantly associated with up- or down-regulation of mRNA expression [21]. Thus, our study emphasizes once again that cultured PBMC is a sensitive and valuable mean for investigation of immunological alterations in MG.

In conclusion, the findings suggested that MuSK-MG is preferentially associated with increased production of IFN-γ, IL-17 and IL-21. Relatively higher IL-21 levels in stimulated PBMC supernatants of AChR-MG patients may indicate a possible role for Tfh cells in MG. Although IS-treatment may have beneficial effects on MuSK-MG and AChR-MG with different mechanisms, in AChR-MG, IS-treatment appears to be preferentially down-regulating Th1 type immune responses. To characterize cytokine patterns better in MuSK-MG and AChR-MG will be recommended for potential future cytokine-specific therapies designed for MG.

## Supporting Information

S1 Table Primer sequences used in RT-PCR. (DOCX)

S2 Table Plasma levels of the measured cytokines. (DOCX)

S3 Table Relative expression of various parameters in CD4^+^ T cells. (DOCX)

S1 File Supporting data of the cytokine measurements of the PBMC. (XLSX)
