# Supplementary material for: Differential Cytokine Changes in Patients with Myasthenia Gravis with Antibodies against AChR and MuSK
Source: PLoS One. 2015 Apr 20;10(4):e0123546. doi: 10.1371/journal.pone.0123546 (PMC4403992; doi:10.1371/journal.pone.0123546)
Supplement: S1 Table — (DOCX) [file pone.0123546.s001.docx]

Supplementary Table 1: Primer sequences used in RT-PCR.

| GENE NAME |  |
| --- | --- |
| *TBET* | F5’-CACCTGTTGTGGTCCAAGTTTA-3’  R5’-GGCCACAGTAAATGACAGGAAT-3’ |
| *GATA3* | F5’-GGAGGTGGATGTGCTTTTTAACA-3’  R5’-ACCTGGCTCCCGTGGTG-3’ |
| *RORG* | F5’-AGGAAGTGACTGGCTACCAGAGG-3’  R5’-GAACTCCACCACGTACTGAATG-3’ |
| *PRDM1* | F5’-AAGGCCAGTGAAGCAGAGA-3’  R5’-CCGATAGGCCATGATGTCT-3’ |
| *IFNG* | F5’-AAACGAGATGACTTCGAAAAGC-3’  R5’-CAGTTCAGCCATCACTTGGAT-3’ |
| *IL10* | F5’-TTCCCTGTGAAAACAAGAGC-3’  R5’-TCACTCATGGCTTTGTAG-ATGC-3’ |
| *IL21* | F5’-TGTTCCCATGCCTTCACCA-3’  R5’-TTTGTCTGGCCTTCTGGAGC-3’ |
| *IL17A* | F5’-CTCATTGGTGTCACTGCTACTG-3’  R5’-CCTGGATTTCGTGGGATTGTG-3’ |
| *CD40L* | F5’-AGATGATTGGGTCAGCACTTTT-3’  R5’-TTCTCCTGTGTTGCATCTCTGT-3’ |
| *GAPDH* | F5’-GCCATCAATGACCCC-TTCATT-3’  R5’-TTGACGGTGCCATGGAATTT-3’ |
