# Supplementary material for: Differential Cytokine Changes in Patients with Myasthenia Gravis with Antibodies against AChR and MuSK
Source: PLoS One. 2015 Apr 20;10(4):e0123546. doi: 10.1371/journal.pone.0123546 (PMC4403992; doi:10.1371/journal.pone.0123546)
Supplement: S2 Table — (DOCX) [file pone.0123546.s002.docx]

Supplementary Table 2: Plasma levels of the measured cytokines and IP-10 in AChR-MG, MuSK-MG and control (CON) groups (pg/ml).

|  | | AChR-MG | | MuSK-MG | | | | CON | | | |  |
| --- | --- | --- | --- | --- | --- | --- | --- | --- | --- | --- | --- | --- |
|  | **Number of samples** | | **Mean±SD** | **Number of samples** | | **Mean±SD** | | **Number of samples** | | **Mean±SD** | | |
| IFN-γ | | 41 | 23,5±14,3 | 24 | 22,1±7,6 | | 42 | | 26,6±12,5 | |  |  |
| IL-10 | | 41 | 7,9±2,5 | 24 | 9±4,2 | | 42 | | 9,1±3,7 | |  |  |
| IL-12p40 | | 41 | 3,8±3 | 24 | 2,4±2,4 | | 42 | | 3±1,8 | |  |  |
| IL-13 | | 41 | 1,2±0 | 24 | 1,2±0 | | 42 | | 1,3±0,3 | |  |  |
| IL-17A | | 32 | 152±372 | 20 | 79±78 | | 37 | | 148±375 | |  |  |
| IP-10 | | 41 | 167±120 | 24 | 159±146 | | 42 | | 130±64 | |  |  |
