# Supplementary material for: Differential Cytokine Changes in Patients with Myasthenia Gravis with Antibodies against AChR and MuSK
Source: PLoS One. 2015 Apr 20;10(4):e0123546. doi: 10.1371/journal.pone.0123546 (PMC4403992; doi:10.1371/journal.pone.0123546)
Supplement: S3 Table — (DOCX) [file pone.0123546.s003.docx]

Supplementary Table 3: Relative expression of various parameters in CD4^+^ T cells of AChR-MG, MuSK-MG patients and healthy controls (CON) as assessed by real time PCR.

|  | AChR-MG | MuSK-MG | CON |
| --- | --- | --- | --- |

| **Gene** | **Number of samples** | **Mean±SD** | **Number of samples** | **Mean±SD** | **Number of samples** | **Mean±SD** |
| --- | --- | --- | --- | --- | --- | --- |
| *IL10* | 23 | 0.2±0.2 | 18 | 0.2±0.1 | 20 | 0.1±0.1 |
| *IFNG* | 24 | 2±5.6 | 19 | 1.2±1.8 | 20 | 1.4±2 |
| *CD40L* | 24 | 11.4±7.5 | 19 | 11.7±6.7 | 20 | 16.2±5.4 |
| *IL17A* | 11 | 0±0 | 10 | 0±0 | 15 | 0±0.1 |
| *IL21* | 14 | 0±0 | 13 | 0±0 | 17 | 0±0 |
| *TBET* | 14 | 0.9±0.5 | 13 | 0.9±0.8 | 17 | 1.4±1 |
| *RORC* | 14 | 1±0.6 | 13 | 1.3±1.2 | 16 | 1.4±0.7 |
| *GATA3* | 13 | 7.8±3 | 13 | 8.3±4.2 | 17 | 10±3.6 |
| *PRDM1* | 13 | 3.5±2.2 | 12 | 2.2±0.9 | 17 | 2.2±1.1 |
